# Proximity proteomics of synaptopodin provides insight into the molecular composition of the spine apparatus of dendritic spines

**DOI:** 10.1073/pnas.2203750119

**Published:** 2022-10-10

**Authors:** Hanieh Falahati, Yumei Wu, Vanessa Feuerer, Hans-Georg Simon, Pietro De Camilli

**Affiliations:** ^a^Department of Neuroscience, Yale University School of Medicine, New Haven, CT 06510;; ^b^Department of Cell Biology, Yale University School of Medicine, New Haven, CT 06510;; ^c^HHMI, Yale University School of Medicine, New Haven, CT 06510;; ^d^Program in Cellular Neuroscience, Neurodegeneration, and Repair, Yale University School of Medicine, New Haven, CT 06510;; ^e^Department of Pediatrics, Feinberg School of Medicine, Northwestern University and Stanley Manne Children’s Research Institute, Chicago, IL 60611

**Keywords:** spine apparatus, dendritic spine, iBioID, in vivo proximity proteomics, Pdlim7

## Abstract

The spine apparatus is a specialized compartment of the neuronal smooth endoplasmic reticulum (ER) located in a subset of dendritic spines. It consists of stacks of ER cisterns that are interconnected by an unknown dense matrix and are continuous with each other and with the ER of the dendritic shaft. While this organelle was first observed over 60 y ago, its molecular organization remains a mystery. Here, we performed in vivo proximity proteomics to gain some insight into its molecular components. To do so, we used the only known spine apparatus–specific protein, synaptopodin, to target a biotinylating enzyme to this organelle. We validated the specific localization in dendritic spines of a small subset of proteins identified by this approach, and we further showed their colocalization with synaptopodin when expressed in nonneuronal cells. One such protein is Pdlim7, an actin binding protein not previously identified in spines. Pdlim7, which we found to interact with synaptopodin through multiple domains, also colocalizes with synaptopodin on the cisternal organelle, a peculiar stack of ER cisterns resembling the spine apparatus and found at axon initial segments of a subset of neurons. Moreover, Pdlim7 has an expression pattern similar to that of synaptopodin in the brain, highlighting a functional partnership between the two proteins. The components of the spine apparatus identified in this work will help elucidate mechanisms in the biogenesis and maintenance of this enigmatic structure with implications for the function of dendritic spines in physiology and disease.

## Significance

The spine apparatus is a specialization of the neuronal endoplasmic reticulum in dendritic spines consisting of stacks of interconnected cisterns that are separated by a dense matrix. Although the spine apparatus has fascinated scientists since 1959, its molecular architecture and precise function remain a major open question in neuroscience. This work addresses the main challenge in studying the spine apparatus, which is the lack of molecular understanding of this organelle, by utilizing an in vivo spatial proteomics technique. Using this approach, we identify proteins associated with the spine apparatus and validate the specific localization of a subset of them, including Pdlim7, in neurons. The results of this work allow for better understanding of brain function in health and disease.

**Fig. 1.**
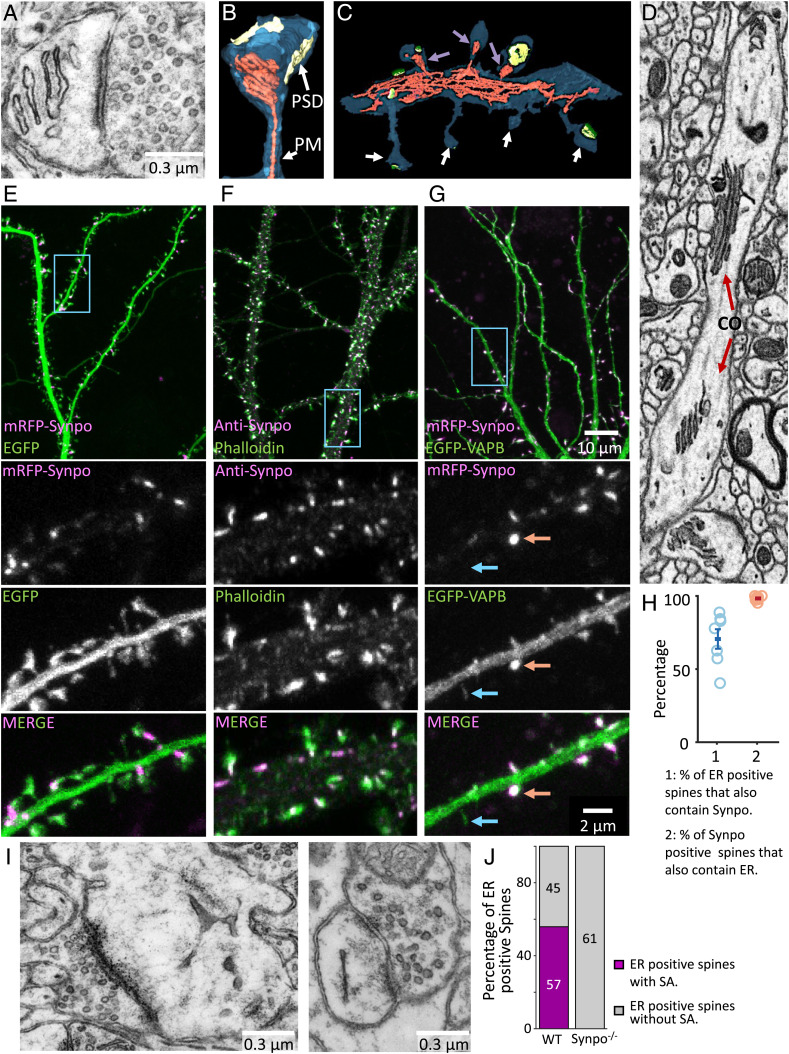
The localization of synaptopodin (indicated as Synpo in all figures) in cultured hippocampal neurons overlaps with the localization of the SA in dendritic spines of cortical slices. (*A*) SA as visualized by transmission EM. (*B* and *C*) SA and ER reconstructed by a semiautomated algorithm from 3D volumes acquired by FIB-SEM. An SA is shown in *B*, while *C* shows a portion of a dendritic shaft with spines containing (magenta arrows) and not containing (white arrows) an SA. The plasma membrane (PM) is shown in blue, the ER is in red, and the post-synaptic density (PSD) is in yellow. (*D*) Cisternal organelle (CO), as observed in an FIB-SEM optical section, at an axonal initial segment. Note that the stacks of ER cisterns are similar to those characteristics of the SA. (*E*) mRFP-synaptopodin coexpressed with cytosolic EGFP as a marker of the entire dendritic volume. Note in the zoomed-in views of the region enclosed by a rectangle (the lower three fields) that synaptopodin is concentrated near the spine neck, where the SA is localized. (*F*) Localization by immunofluorescence of endogenous synaptopodin showing strong overlap with a pool of F-actin labeled by phalloidin-Alexa488. Also, in this sample, the magnified views (the lower three fields) show enrichment of synaptopodin, relative to actin, at the spine neck. (*G*) mRFP-synaptopodin coexpressed with an ER marker, EGFP-VAPB, showing colocalization of the two proteins. In the zoomed-in views (the lower three fields), red arrows show a spine positive for both the ER marker and synaptopodin, and the blue arrows show a spine with the ER marker but lacking synaptopodin. (*H*) Percentage of ER-positive spines that also contain synaptopodin and percentage of synaptopodin-positive spines that contain ER quantified in cultured hippocampal neurons expressing mRFP-synaptopodin and the ER markers EGFP-VAPB or EGFP-Sec61*β*. Each data point represents at least 99 spines from a single neuron. (*I*) Spine of a synaptopodin KO mouse that lacks the SA but contains the ER. (*J*) Quantification of the number of spines where ER was visible in the plane of the section with or without an SA in the brain of wild type (WT) vs. synaptopodin mutant mice (n≥800 spines per genotype).

The neuronal endoplasmic reticulum (ER) is an intricate continuous network of membrane tubules and cisterns that runs throughout neuronal processes with region-specific specializations. One such specialization of the smooth ER is the spine apparatus (SA) that is located in a subset of dendritic spines. The SA consists of stacks of flat cisterns that are connected by an unknown dense matrix and are continuous with each other and with the ER of the dendritic shaft (1–3) (Fig. 1*A* and *SI Appendix*, Fig. S1*A*). Morphological changes in the SA have been reported after long-term potentiation (4) and also, in a variety of human disorders, including several neurodegenerative conditions (5–9). While the first observation of the SA by electron microscopy (EM) was reported in 1959 by Gray (1), our understanding of this organelle remains fairly limited. Its molecular characterization has proven to be challenging due to the difficulty of its biochemical isolation and its absence in organisms suitable for genetic screens.

The only known protein enriched at the SA and required for its formation is synaptopodin, a protein without transmembrane regions localized in the cytosolic space (10). Neuronal synaptopodin specifically localizes to dendritic spines and to the axonal initial segment, where another specialization of the ER similar to the SA (stack of flattened cisterns) called the cisternal organelle is present (11–14). Lack of synaptopodin in synaptopodin knock-out (KO) mice correlates with the lack of SA and of the cisternal organelle, as well as with a reduction in Hebbian plasticity and spatial memory (11, 15–18). A longer isoform of synaptopodin is expressed in the foot processes of podocytes, where it functions as a regulator of the actin cytoskeleton (19, 20). Synaptopodin binds to and bundles actin (21) and interacts with several actin binding proteins, such as *α*-actinin (13, 21, 22). While more is known about the interactors of synaptopodin in podocytes, its binding partners at the SA remain unknown.

The goal of this work was to gain insight into the molecular composition of the SA. To this aim, we used synaptopodin as a starting point for our analysis. We identified some of its binding partners by an in vivo proximity biotinylation approach and characterized the specific localization of a subset of these proteins in neurons and their interaction with synaptopodin in an exogenous system.

## Results

### Localization of Synaptopodin Correlates with the Presence of the SA in Neurons.

As a premise to our molecular analysis, we examined the morphology of the SA by electron microscopy. Representative electron micrographs of the SA are shown in Fig. 1*A* and *SI Appendix*, Fig. S1*A*. Our analysis of three-dimensional (3D) volumes of cerebral cortex in FIB-SEM (focused ion beam scanning EM) volumes reported previously (23) or in serial section scanning electron microscopy (ssSEM) volumes available on MICrONS Explorer (24) shows that between 40 and 60% of all evaluated spines contain the SA (*SI Appendix*, Fig. S1*B*). We defined the SA as a portion of ER located in dendritic spines with two or more closely apposed parallel flat cisterns. In order to further examine the morphology and localization of the SA within spines, we examined images of 3D volumes of mouse cerebral cortex from the FIB-SEM dataset, as this technique allows for better resolution compared with ssSEM (3, 25). We developed an algorithm to semiautomatically detect SAs in FIB-SEM images. A segment of the SA was manually selected in one plane, and its remaining portion was automatically tracked in the other parallel planes. Using this algorithm, we identified and examined 83 SAs in FIB-SEM images of three samples of mouse cerebral cortex reported previously (23) (examples are in Fig. 1 *C* and *D*, Movie S1, and *SI Appendix*, Fig. S1*C*). Typically, the SA was located close to the boundary of the spine head with the spine neck (Fig. 1*B* and *SI Appendix*, Fig. S1 *A*, *C*, and *D*). The ER is known to form contacts with other membranes where ions or lipids are exchanged; 83% of the identified SAs had at least one site of close appositions with the plasma membrane, while 41% of them were in contact with one or more tubulovesicular structures not connected to the ER, most likely endosomes (*SI Appendix*, Fig. S1*D* shows examples). The number of ER cisterns within SAs varied between two and eight, with the median number being three (*SI Appendix*, Fig. S1*E*). A dense matrix was present not only between cisterns but also, in some cases, at the free surface of one of the two outer cisterns of the SA (Fig. 1*A* and *SI Appendix*, Fig. S1*A*). As expected, analysis of FIB-SEM 3D volumes also revealed stacks of ER cisterns similar to those of the SAs but more extended in width, at axonal initial segments where they are called cisternal organelles (Fig. 1*D*).

Inspection of hippocampal neurons in primary cultures expressing synaptopodin tagged with monomeric red fluorescent protein (mRFP-synaptopodin) and the cytosolic marker EGFP (enhanced green fluorescent protein) confirmed the presence of synaptopodin in 33 ± 8% of spines, where it strongly colocalized with a pool of F-actin as detected by phalloidin (Fig. 1 *E* and *F* and *SI Appendix*, Fig. S1*F*) (12). Consistent with EM observations, mRFP-synaptopodin was concentrated close to the neck of the spine (the high-magnification fields in Fig. 1*E*), where a stable pool of F-actin is reported to reside, and was absent from the head where branched F-actin predominates (Fig. 1*F*) (26). In addition, in a subset of cultured hippocampal neurons, endogenous synaptopodin localized to the axonal initial segment where the cisternal organelle is observed by EM (*SI Appendix*, Fig. S1*G*) (11, 27). In agreement with synaptopodin being a component of the SA, mRFP-synaptopodin fluorescence closely overlapped with the fluorescence of the ER markers EGFP-VAPB or EGFP-Sec61*β* (28, 29), with 98% of the spines positive for synaptopodin also being positive for these ER markers (Fig. 1 *G* and *H*). In contrast, 40 ± 8% of the total number of spines contained ER, and of those, only 71% also contained synaptopodin puncta (Fig. 1 *G* and *H* and *SI Appendix*, Fig. S1*F*). Similar statistics were reported for organotypic slices (30–32).

Synaptopodin is necessary for the stacking of SA cisterns as previously reported by EM in the brain tissue of synaptopodin KO mice (15) and as confirmed by us in these mice (Fig. 1 *I* and *J*). However, it is clearly not required to recruit ER to spines as cultured hippocampal neurons of synaptopodin KO mice contained ER, as detected by the expression of dsRed-KDEL in a subset of spines (*SI Appendix*, Fig. S1*H*). EM images further showed that ER cisterns can be present in these spines, although stacks do not form (Fig. 1 *I* and *J*) (15). These findings are consistent with a scenario in which synaptopodin is part of the dense cytoskeletal matrix that connects to each other cisterns of the SA.

### Identification of the SA Protein by Proximity Labeling.

**Fig. 2.**
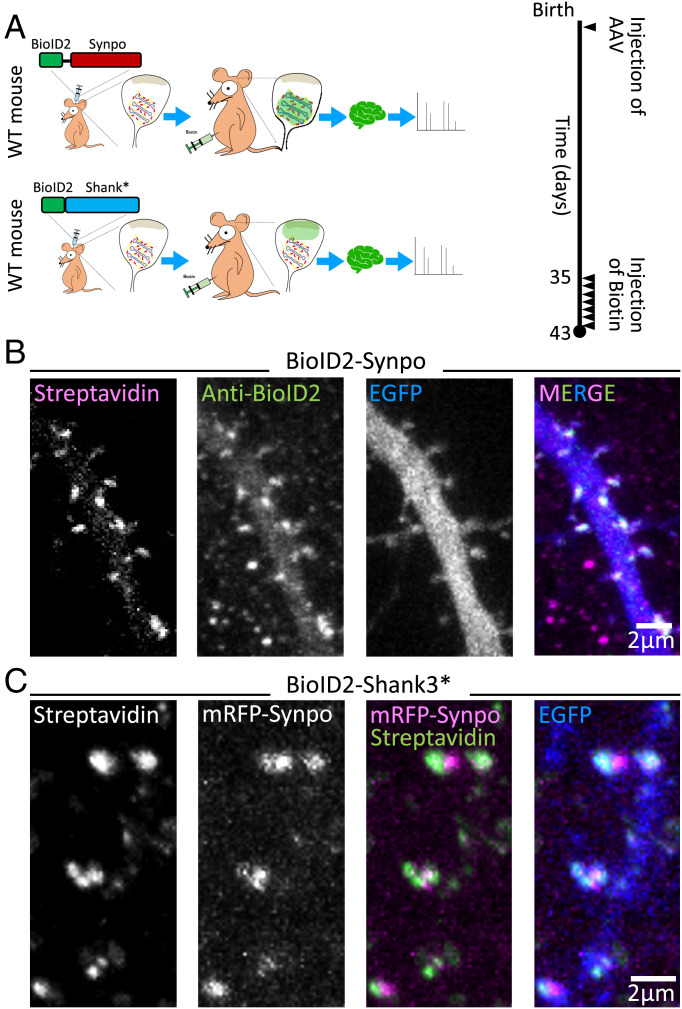
Identification of SA-associated proteins by in vivo proximity labeling. (*A*) Schematic representation of in vivo proximity labeling of protein neighbors of synaptopodin (iBioID). To target the biotinylating enzyme to the SA, AAV2/9 viruses containing BioID2-synaptopodin were injected in the cortex of neonatal mice. BioID2 fused to a fragment of Shank3 (BioID-Shank3*) was used as a control to localize the biotinylating enzyme to a different region of the spine (i.e., the neighborhood of the PSD). Biotin was administered intraperitoneally starting 5 wk after birth for 7 consecutive days. On day 8, the mouse was euthanized, and biotinylated proteins were isolated from their brain. WT: wild type. (*B*) Cultured hippocampal neurons transfected with BioID2-synaptopodin and EGFP were stained with Alexa647-streptavidin and anti-BioID2 antibody to confirm the specificity of biotinylation in spines. (*C*) A cultured hippocampal neuron expressing mRFP-synaptopodin, BioID2-Shank3*, and EGFP was labeled with streptavidin to examine the specificity of biotinylation as well as the differential localization of synaptopodin and of the control protein Shank3* in spines.

To identify components of the SA that may cooperate with synaptopodin in forming this structure, we used an in vivo proximity-labeling approach (Fig. 2*A*) (33). We generated a fusion protein of synaptopodin and BioID2 (34) (BioID2-synaptopodin) and confirmed that this protein, when expressed in hippocampal cultured neurons, was targeted to dendritic spines by anti-BioID2 immunofluorescence (Fig. 2*B*). Moreover, streptavidin labeling confirmed the presence of biotinylated signal overlapping with BioID2 immunoreactivity, confirming the occurrence of local biotinylation. Next, AAV2/9 (adeno-associated virus2/9) viruses containing this construct were injected in the cerebral cortices of neonatal mice, and after 5 wk, the substrate, biotin, was administered through intraperitoneal injection for 7 consecutive days (Fig. 2*A*). At the end of the 6 wk, mice were euthanized, extracts of the cerebral cortices were prepared, and biotinylated proteins were purified from these extracts using streptavidin beads and identified by mass spectrometry. As a control, in parallel experiments, a fusion protein of BioID2 and Shank3* (a fragment of Shank3 comprising amino acids 1,055 to 1,806 to make it packageable in AAV) (Fig. 2*A*) was expressed in mouse cerebral cortices by the same procedure. Like synaptopodin, Shank3* is an actin-associated protein that is enriched in dendritic spines but concentrated in the subsynaptic region rather than close to the spine neck where the synaptopodin is localized. Accordingly, streptavidin labeling of cells expressing BioID2-Shank3* revealed that the biotin signal generated by this construct was juxtaposed, rather than precisely colocalized, with mRFP-synaptopodin (Fig. 2*C*). Thus, comparison of the proteome of BioID2-synaptopodin and BioID2-Shank3* is expected to allow for spatial mapping of the dendritic spine and to identify proteins enriched in proximity of synaptopodin. We performed two separate experiments in which brain extract of multiple pups was collected and results were statistically analyzed (*Materials and Methods* has details).

We identified 140 proteins enriched with statistical significance in the BioID2-synaptopodin proteome relative to the Shank3* proteome (*SI Appendix*, Fig. S2). These included a large number of signaling and scaffold proteins as well as actin-related proteins likely comprising structural components of the SA as well as “client” and regulatory proteins of the SA. They included, as expected, many ER components, such as proteins involved in calcium storage and signaling (e.g., Ryr2, Ip3r3/Itpr3, and Stim1), lipid transfer (e.g., Pitpnm1-3, Osbpl, and TMEM24/C2cd2l), and lipid metabolism [for example, Faah, a protein implicated in endocannabinoid metabolism and previously reported to be localized in spines (35)]. As expected, the BioID2-Shank3* control proteome was more enriched in proteins of postsynaptic densities (PSDs), such as Shank1-2 and Homer1-3, and receptors for glutamate, including the N-methyl-D-aspartate (NMDA) receptors Grin1, Grin2a, and Grin2b and the *α*-amino-3-hydroxy-5-methyl-4-isoxazolepropionic acid (AMPA) receptors Gria2 and Gria3 as well as the metabotropic receptors Grm2 and Grm5 (Dataset S1). The difference between the two proteomes was further supported by the analysis of Gene Ontology terms for the identified proteins. The highest enriched cellular components for BioID2-synaptopodin samples included ER tubular network and cortical ER, while the enriched gene ontology terms for BioID2-Shank3* samples included anchored and cytosolic components of PSDs. Even the actin-associated proteins enriched in the two proteomes were different, as BioID2-synaptopodin samples were enriched for contractile actin and stress fiber components (e.g., *α*-actinin-1, *α*-actinin-2, *α*-actinin-4, and Myosin-9), while BioID2-Shank3* samples contained components of Arp2/3 and SCAR/WAVE (Suppressor of Cyclic AMP Receptor mutation/Wiskott Aldrich VErprolin homologous protein) complexes (e.g., Arpc1a, Arpc2, Arpc5l, Wasf3, Abi1-2, Nckap1, and Cyfip1-2).

### Colocalization of Identified Proteins with Synaptopodin in Dendritic Spines.

**Fig. 3.**
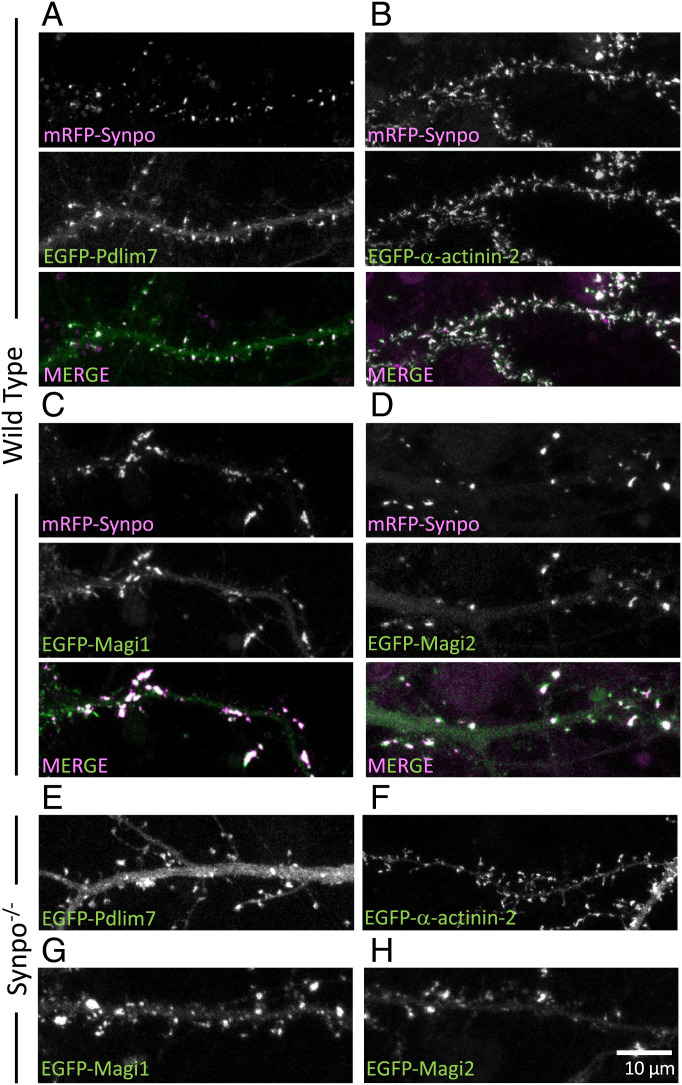
Colocalization with synaptopodin in dendritic spines of proteins identified by our screen. (*A*–*D*) Cultured wild-type hippocampal neurons expressing mRFP-synaptopodin with EGFP-Pdlim7 (*A*), EGFP-*α*-actinin-2 (*B*), EGFP-Magi1 (*C*), or EGFP-Magi2 (*D*). (*E*–*H*) Cultured hippocampal neurons of synaptopodin KO mice transfected with EGFP-Pdlim7 (*E*), EGFP-*α*-actinin-2 (*F*), EGFP-Magi1 (*G*), or EGFP-Magi2 (*H*).

To validate our biochemical findings, we investigated whether a selected few identified proteins are indeed localized in close proximity of synaptopodin in situ. We focused on proteins with a proposed or proven link to synaptopodin, such as Pdlim7 (a member of the PDZ (post synaptic density protein [PSD95], Drosophila disc large tumor suppressor [Dlg1], and zonula occludens-1 protein [zo-1]) and LIM (Lin-11, Islet-1, and Mec-3) domain protein family and a potential interactor reported in Biogrid), MAGUK inverted 1 (Magi1; a member of the membrane-associated guanylate kinases (MAGUK) protein family that comprises WW, PDZ, and GK (guanylate kinase) domains) (20), *α*-actinin-2, *α*-actinin-4 (21, 22), and Magi2 (a putative synaptopodin interactor because of its similarity to Magi1) (36), and we expressed these proteins with a fluorescent tag in cultured hippocampal neurons. We found that in dendritic spines, EGFP-Pdlim7 precisely colocalized with mRFP-synaptopodin (Fig. 3*A*). Strong overlap was observed in spines between synaptopodin, EGFP-*α*-actinin-2, EGFP-Magi1, and EGFP-Magi2 (synaptic scaffolding proteins, S-SCAM) (Fig. 3 *B* and *C*). The different appearances of spines upon exogenous expression of Magi1 and Magi2 proteins (fewer and larger) and of *α*-actinin-2 (thinner and longer) are in agreement with previous reports (37, 38). However, synaptopodin had no detectable effect on the localization of EGFP-Magi1/2 and EGFP-*α*-actinin-2 in dendritic spines, as these proteins still localized in spines in synaptopodin KO hippocampal cultured neurons (Fig. 3 *F*–*H*). In contrast, in synaptopodin KO neurons, an obvious greater diffuse fluorescent signal from EGFP-Pdlim7 throughout dendrites was observed (Fig. 3*E*). The enrichment of Pdlim7 in spines relative to dendritic shafts in wild-type neurons was twice that observed in synaptopodin KO neurons (5.1- ± 0.3-fold vs. 2.4- ± 0.3-fold) (*SI Appendix*, Fig. S3*A*). This suggests that while Pdlim7 can localize to spines independently of synaptopodin (probably through its binding to F-actin), synaptopodin significantly enriches Pdlim7 in spines, pointing to a special relation between synaptopodin and Pdlim7. Pdlim7 was also found at axon initial segments, where the cisternal organelle is localized (*SI Appendix*, Fig. S3*B*), with the same localization pattern as synaptopodin, further strengthening a partnership between these two proteins. Pdlim7 is not necessary for the formation of the SA, as such an apparatus can still be observed in previously generated Pdlim7 KO mice (39) (*SI Appendix*, Fig. S3*C*).

### Interaction of Synaptopodin with Pdlim7, Magi1, Magi2, and α-actinin-2 as Assessed by Expression in Nonneuronal Cells.

**Fig. 4.**
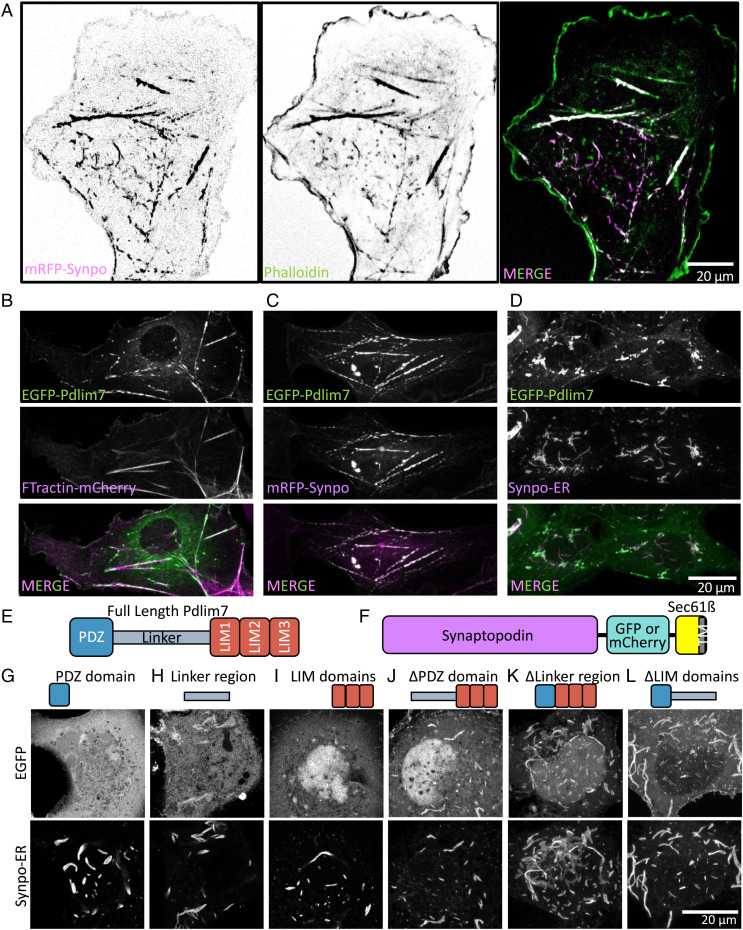
Pdlim7 interacts with synaptopodin in COS-7 cells. (*A*) COS-7 cells expressing mRFP-synaptopodin alone show strong overlap of the mRFP fluorescence with phalloidin staining. (*B*) Similar to synaptopodin, EGFP-Pdlim7 partially colocalizes with FTractin-mCherry on stress fibers when expressed alone in COS-7 cells. (*C*) mRFP-synaptopodin precisely colocalizes with EGFP-Pdlim7. (*D*) Coexpression of synaptopodin-ER with EGFP-Pdlim7 results in recruitment of Pdlim7 to synaptopodin assemblies in the ER. (*E*) Domain structure of Pdlim7. (*F*) Schematic structure of synaptopodin-ER, which is made by fusion of synaptopodin to the ER protein, Sec61*β*. TM: transmembrane. (*G*–*L*). Coexpression of the Pdlim7 fragments shown with synaptopodin-ER. All Pdlim7 constructs were tagged with EGFP at the N terminus. All constructs containing the linker region colocalize with synaptopodin-ER. While the PDZ domain or the LIM domains alone show little colocalization with synaptopodin-ER, a construct comprising both regions interacts with synaptopodin even in the absence of the linker region. These data demonstrate the multivalency in the interaction between synaptopodin and Pdlim7.

We further investigated the relation of Pdlim7, Magi1, Magi2, and *α*-actinin-2 to synaptopodin by expressing them in nonneuronal cells, as the lack of endogenous synaptopodin (40) and the large size of these cells allow us to assess more precisely protein colocalizations. In these cells, mRFP-synaptopodin colocalized with filamentous actin as visualized by phalloidin staining as described previously (10), although with variable relative intensity (Fig. 4*A*). Similar to synaptopodin and in agreement with the reported role of Pdlim7 in the regulation of the actin cytoskeleton (39, 41), EGFP-Pdlim7 was also associated with actin stress fibers when expressed alone in COS-7 cells, as indicated by its colocalization with FTractin-mCherry (Fig. 4*B*), and showed a discontinuous localization on them, as observed for synaptopodin (Fig. 4*A*). When coexpressed with mRFP-synaptopodin, EGFP-Pdlim7 precisely colocalized on all structures positive for this protein (Fig. 4*C*). We further investigated the (direct or indirect) interaction of the two proteins by determining whether ectopically targeting synaptopodin to the ER would result in a parallel relocalization of Pdlim7 with synaptopodin to the ER. To this aim, we generated a chimera comprising in sequence synaptopodin, a fluorescence protein, and Sec61*β*, an ER resident protein anchored to this organelle by a C-terminal transmembrane region (29) (Fig. 4*F*). This chimera, referred to henceforth as synaptopodin-ER, resulted in the formation of large linear assemblies most likely reflecting clusters of synaptopodin along ER elements (*SI Appendix*, Fig. S4*A*). When coexpressed with synaptopodin, EGFP-Pdlim7 also relocated to these assemblies (Fig. 4*D*), confirming a direct or indirect interaction of the two proteins. Interestingly, such assemblies were also positive for phalloidin (*SI Appendix*, Fig. S4*A*), indicating that synaptopodin can nucleate or recruit F-actin at the ER surface.

As Pdlim7 is a newly identified component of spines and is the protein that precisely colocalizes with synaptopodin in both neurons and fibroblasts, we examined in more detail its interaction with synaptopodin. Coexpression of Pdlim7 deletion constructs with synaptopodin revealed that all constructs containing the linker region, including the linker region alone, robustly colocalized with synaptopodin-ER (Fig. 4 *H*, *J*, and *L*). However, while the LIM domain region alone (Fig. 4*I*) and the PDZ domain alone (Fig. 4*G*) were primarily cytosolic or nuclear, respectively, a construct comprising the LIM domains and the PDZ domain but lacking the linker region also colocalized strongly with synaptopodin (Fig. 4*K*). We conclude that interactions involving multiple domains of Pdlim7 (i.e., a strong interaction with the linker region and weak interactions that function cooperatively within the PDZ and the LIM domains) drive its coassembly with synaptopodin.

**Fig. 5.**
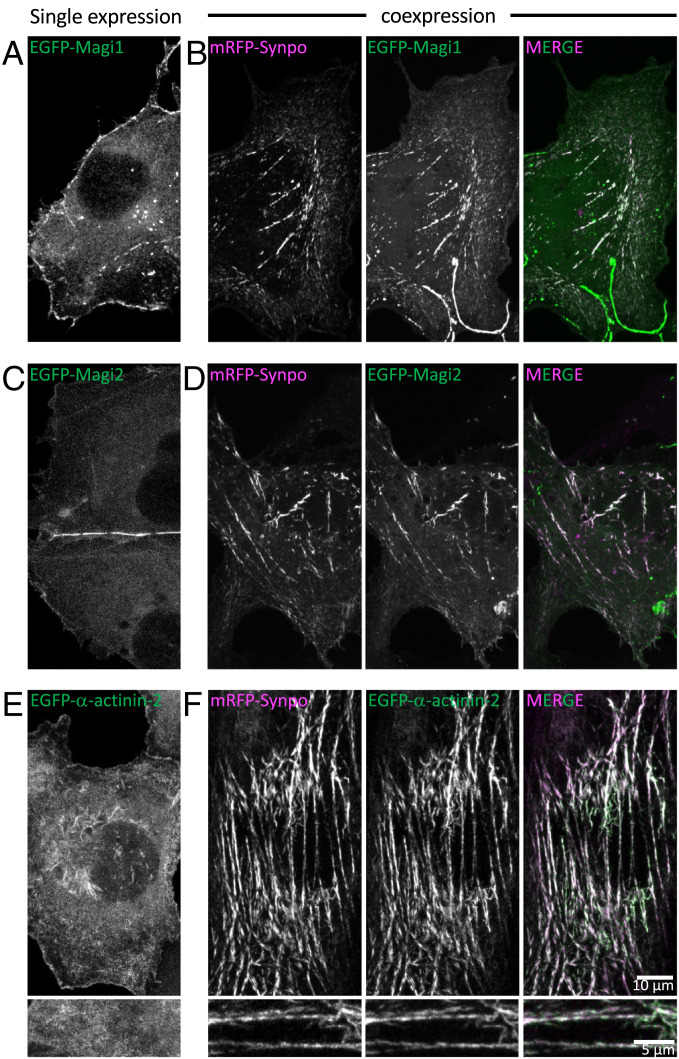
Proteins identified by our screen colocalize with synaptopodin in COS-7 cells. (*A*–*D*) EGFP-Magi1 and EGFP-Magi2 localize under the cell surface and primarily at cell–cell junctions when expressed alone in COS-7 cells. Both proteins partially relocalize to synaptopodin-positive structures when expressed together with mRFP-synaptopodin. (*E* and *F*) EGFP-*α*-actinin-2 localizes under the cell surface and in the cytosol when expressed alone in COS-7 cells, but it relocates to synaptopodin-positive structures when coexpressed with mRFP-synaptopodin. *E*, *Lower* and *F*, *Lower* show high-magnification views.

EGFP-Magi1 and EGFP-Magi2 localized under the plasma membrane with an enrichment at cell–cell junctions when expressed in COS-7 cells alone (Fig. 5 *A* and *C*). However, upon coexpression with synaptopodin, these two proteins were also recruited to synaptopodin-positive internal structures (Fig. 5 *B* and *D*). Similarly, expression of the ER-targeted chimera (synaptopodin-ER) resulted in the relocation of Magi1 and Magi2 to synaptopodin assemblies at ER (*SI Appendix*, Fig. S4 *B* and *C*). Synaptopodin is also required for the recruitment of *α*-actinin-2 to actin filaments in COS-7 cells since when expressed alone, EGFP-*α*-actinin-2 localized under the plasma membrane and in the cytosol but relocated to synaptopodin assemblies upon coexpression with mRFP-synaptopodin (Fig. 5 *E* and *F*) or synaptopodin-ER (*SI Appendix*, Fig. S4*D*).

**Fig. 6.**
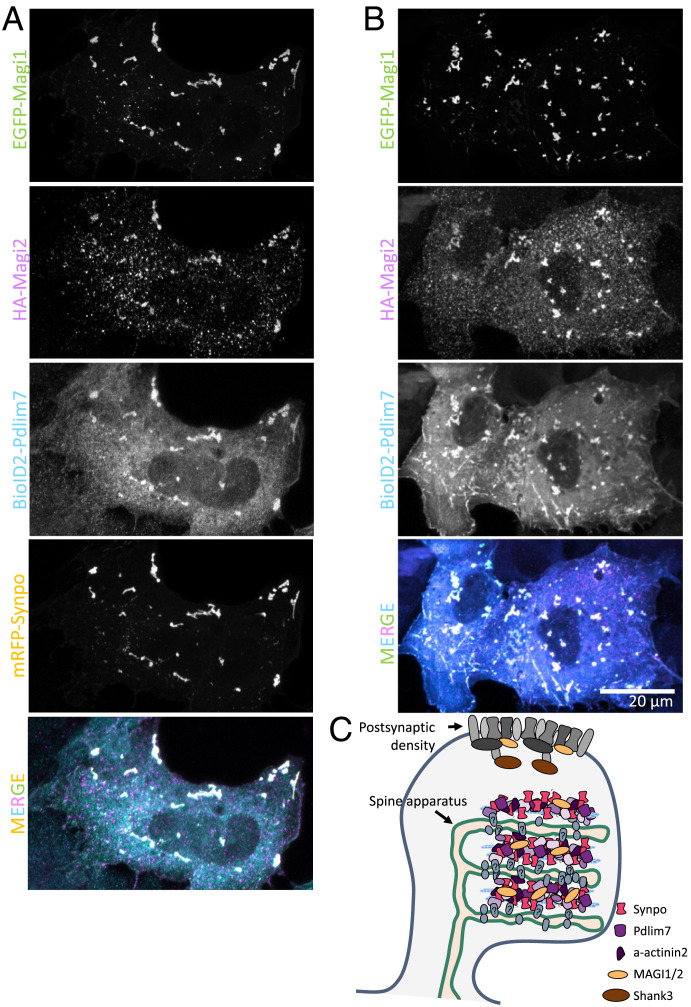
Proteins identified by our screen coassemble. (*A*) When mRFP-synaptopodin, EGFP-Magi1, HA-Magi2 (labeled with anti-HA antibody), and BioID2-Pdlim7 (labeled with anti-BioID2 antibody) are coexpressed in COS-7 cells, they all colocalize. (*B*) Even in the absence of synaptopodin, Pdlim7 can recruit Magi1 and Magi2 to internal structures. (*C*) Schematic representation of a spine showing the SA-associated proteins that were identified and validated in this study. HA: epitope tag from hemagglutinin.

Finally, when synaptopodin, Magi1, Magi2, and Pdlim7 were all coexpressed together in COS-7 cells, they all coassembled into the same structures (Fig. 6*A*). Interestingly, synaptopodin was not necessary for this coassembly, as BioID2-Pdlim7 was sufficient to recruit Magi1 and Magi2 (Fig. 6*B*). These results underscore the multivalency of the interactions among identified SA-associated proteins.

### Pdlim7 Has an Expression Pattern Similar to Synaptopodin.

Proteins that are part of the same structure or cooperate in their function may have similar patterns of expression. The expression pattern of synaptopodin varies depending on the neuronal population. Its highest level of expression is in cortical and hippocampal neurons where the SA is also detectable, while neurons, such as Purkinje cells, that lack the SA, despite having a high abundance of dendritic spines, do not express synaptopodin. We, therefore, examined how similar the expression pattern of Pdlim7 was to synaptopodin in a previously reported single-cell RNA sequencing database, DropViz (42). We quantified the similarity in the expression pattern by calculating the Pearson correlation coefficient, *r*, between the number of reads in every cell for any gene of interest (GoI), including Pdlim7, and that of synaptopodin using the following equation:

[1] $$r_{N_{\mathrm{Synpo}},N_{\mathrm{GoI}}}=\frac{E[(N_{\mathrm{Synpo}}-\mu_{N_{\mathrm{Synpo}}})(N_{\mathrm{GoI}}-\mu_{N_{\mathrm{GoI}}})]}{\sigma_{N_{\mathrm{Synpo}}}\sigma_{N_{\mathrm{GoI}}}},$$

where *N_Synpo_* and *N_GoI_* are the numbers of reads of synaptopodin and GoI in each cell, respectively; *E* is the expected value; $\mu_{N_{\mathrm{Synpo}}}$ and $\mu_{N_{\mathrm{GoI}}}$ are the means of reads in each cell for synaptopodin and for every gene, respectively; and $\sigma_{N_{\mathrm{Synpo}}}$ and $\sigma_{N_{\mathrm{GoI}}}$ are the SDs of *N_Synpo_* and *N_GoI_*, respectively. While the correlation values vary between –0.04 and 0.558, Pdlim7 scores 0.31 and ranks among the top genes with the highest correlation with synaptopodin. This underlines the functional association between synaptopodin and Pdlim7.

## Discussion

While the SA has been observed for over 60 y, the mechanism of its formation and function remains elusive mainly due to the lack of a comprehensive understanding of its molecular composition. More specifically, the nature of the dense matrix connecting ER cisterns remains unknown, besides evidence that it contains the protein synaptopodin, an actin binding protein. We identify 140 proteins with a propensity to localize in proximity of synaptopodin in brain, as assessed by their enrichment in the proximity proteome of BioID2-synaptopodin relative to the proximity proteome of BioID2-Shank3*, a molecular component of the PSDs. We confirmed the colocalization and selective concentration of four of the hits identified by this analysis, Pdlim7, Magi1, Magi2, and *α*-actinin-2, with synaptopodin in neuronal dendritic spines (13, 20–22, 43). Furthermore, we showed that when Pdlim7, Magi1, and Magi2 are expressed together in nonneuronal cells, they coassemble with synaptopodin and actin, supporting the possibility that they may be part of the matrix that connects to each other ER cistern of the SA (Fig. 6*C*). Pdlim7 also showed an expression pattern similar to that of synaptopodin, thus supporting the likely role of Pdlim7 as a critical functional partner of this protein in the SA.

The different proximity proteome of BioID2-synaptopodin and BioID2-Shank3* validates the in vivo approach that we have employed toward the identification of components of the SA and of proteins that may be concentrated in its proximity. As expected, these two proteomes comprise transmembrane proteins but primarily ER proteins in the case of synaptopodin and plasma membrane proteins in the case of Shank3*. Analysis of the synaptopodin proteome reveals a variety of housekeeping proteins of the smooth ER, but none of the most enriched ER proteins that we tested were found to be specifically concentrated in dendritic spines (*SI Appendix*, Table S1). Thus, we have found no evidence that the stacking of ER cisterns in the SA reflects the concentration at these sites of a specific ER protein that interacts with synaptopodin or with another protein of the intervening matrix, although the existence of such protein remains possible. A plausible scenario is that the stacking of ER cisterns via an intervening protein matrix may be due to a multiplicity of low-affinity interactions between components of this matrix and the ER membrane. According to such a scenario, the matrix of the SA would have the property of a liquid protein condensate (44, 45) generated by a multiplicity of low-affinity interactions.

The protein neighbors of synaptopodin that we have validated are all factors implicated in actin-based scaffolds. Pdlim7, an actin regulatory protein known to have an important role in a variety of cell types (39, 46), was a previously unknown component of the SA. While we found that Pdlim7 is not necessary for the formation of the SA, likely due to redundancy with other SA proteins, its striking colocalization with synaptopodin both in neurons and in fibroblasts, as well as the similar pattern of expression of the two proteins in brain, suggests their functional partnership. This partnership likely reflects a specific interaction that can be either direct or indirect. The two proteins are absent from the bulk of ER in neurons but only present and colocalized at dendritic spines and at axonal initial segments. Moreover, the striking selective recruitment of Pdlim7 to ER-targeted synaptopodin (Fig. 4*D*) implies that even if such recruitment was meditated by F-actin bound to synaptopodin, it is specific to the subset of actin filaments that are also associated with synaptopodin. Presence of the PDZ domain in Pdlim7 would be consistent with an interaction with a membrane protein (47), but so far, binding partners for this PDZ domain have not been identified. Most frequently, PDZ domains bind to plasma membrane–localized proteins (47), but it cannot be excluded that Pdlim7 may anchor the matrix to some intrinsic membrane protein of ER cisterns.

Magi proteins Magi1 and Magi2 (S-SCAM) are scaffolding proteins typically localized in the protein matrix that lines the cytosolic leaflet of the plasma membrane, primarily at cell–cell junctions. In the brain, Magi2 was shown to be a structural component of PSDs that regulate the trafficking of NMDA and AMPA receptors (36, 37, 48–50). Thus, the enrichment of Magi2 (S-SCAM) in the BioID2-synaptopodin proteome, relative to the BioID2-Shank3* proteome, was surprising. However, its paralog Magi1 is a known interactor of synaptopodin in kidney podocytes (20), and both Magi1 and Magi2 were shown to play a role in the organization of the glomerular filtration barrier, consistent with the known role of synaptopodin in the generation and maintenance of such barrier (36, 51, 52). The interaction of Magi1 and Magi2 with synaptopodin is supported by our transfection experiments in nonneuronal cells, where both Magi1 and Magi2 localized under the plasma membrane and predominantly at cell–cell contacts when expressed alone but partially relocalized to internal synaptopodin-positive structures when coexpressed with synaptopodin or Pdlim7. The matrix of the SA may contain a reserve pool of these proteins. In fact, one of the functions of the SA may be to act as a reservoir of cytosolic proteins implicated in postsynaptic function.

Although the focus of the present study was to elucidate mechanisms underlying the structure of the SA, the synaptopodin and Shank3* proximity proteome that we have characterized can be mined to learn more about spine cell biology. Our results also showed that the in vivo proximity-labeling approach employed by us and originally used to identify proteins implicated in postsynaptic inhibitory signaling (33) is a very powerful methodology to study subdomains of the dendritic spine. Taken together, the data provided here are a starting point not only for understanding the formation and function of the SA but also, for gaining mechanistic insight into the physiological processes occurring in dendritic spines.

## Materials and Methods

### Electron Microscopy of Brain Tissue.

All experiments were carried out in accordance with NIH guidelines and approved by the Yale IACUC (Institutional Animal Care and Use Committee). All mice were maintained in the vivarium with a 12-h light–dark cycle, stable temperature at 22 ^∘^C ± 1 ${}^{{}^{\circ}}$C, and humidity between 20 and 50%. Wild-type (C57BL/6) and synaptopodin KO mice were obtained from The Jackson Laboratory. Pdlim7 KO mice were reported previously (39) and were generated from heterozygous crosses.

Three-month-old wild-type (C57BL/6), synaptopodin KO, and Pdlim7 KO mice were anesthetized with a ketamin/xylazine anesthetic mixture and transcardially perfused with 2% paraformaldehyde and 2% glutaraldehyde in 0.1 M sodium cacodylate buffer, pH 7.4, at 37 ${}^{{}^{\circ}}$C. Brains were dissected out, kept overnight in the same fixative, subsequently trimmed in small blocks (less than 0.5 × 0.5 × 0.5 mm), kept in fresh 2.5% glutaraldehyde in 0.1 M sodium cacodylate buffer for another hour at room temperature, postfixed in 2% OsO_4_ + 1.5% K_4_Fe(CN)_6_ (Sigma-Aldrich) in 0.1 M sodium cacodylate buffer for 1 h, en bloc–stained in 2% aqueous uranyl acetate for 1 h, dehydrated in increasing concentrations of ethanol, and embedded in EMbed 812. Ultrathin sections (50 to 60 nm) were observed in a Talos L 120C transmission EM microscope at 80 kV. Images were taken with Velox software and a 4K × 4K Ceta CMOS Camera (Thermo Fisher Scientific). Except when noted, all EM reagents were from EMS.

### Semiautomated Detection of SA in FIB-SEM Images.

To morphologically characterize SA in FIB-SEM images, an in-house ImageJ macro was developed and used. First, a difference of Gaussian (DoG) filter was applied to the images to detect the edges of features of interest. To generate a mask, a manually selected threshold was applied to the filtered image. Then, the mask for the feature of interest was selected in one plane manually and tracked automatically in other planes. This automatic tracking takes advantage of the continuity in the structure of the SA. The final mask can also be refined by adding or removing mischaracterized portions of the mask using a similar semiautomated approach. Other subcellular features, such as plasma membrane, PSD, or mitochondria, can be detected with this code by changing the DoG filter.

### In Vivo BioID.

In vivo BioID (iBioID [in vivo proximity-dependent biotin identification]) was performed using the method described previously (33) with some minor modifications. The AAV2/9 viruses containing pAAV-BioID2-synaptopodin or pAAV-BioID2-Shank3* were injected into C57/B6J mouse cortex at postnatal day 0-2. At 5 wk of age, biotin was subcutaneously injected at 24 mg/kg for 7 consecutive days to increase the biotinylation efficiency. Biotinylated proteins were purified from the forebrain of each mouse separately. This experiment was performed on two different litters. To better control for the genetic variability, each round of the experiment included injections of all the pups of the litter. In the first case (experiment 1), four mice were injected with BioID2-synaptopodin, and three mice were injected with BioID2-Shank3*. In the second experiment (experiment 2), five mice were injected with BioID2-synaptopodin, and four mice were injected with BioID2-Shank3*. Biotinylated proteins from each litter were purified using two slightly different methods.

For experiment 1, the forebrains were homogenized on ice using a glass/Teflon homogenizer with 0.5 mL of lysis buffer (50 mM Hepes (N-2-hydroxyethylpiperazine-N’-2-ethanesulfonic acid), pH 7.5, 150 mM NaCl, 1 mM EDTA (ethylenediamine tetraacetic acid), 0.25 µL benzonase, EDTA-free cOmplete mini protease inhibitor mixture; 11836170001; Roche Diagnostics). The samples were then transferred to an Eppendorf tube, and lysis buffer containing 10% sodium dodecyl sulfate (SDS) was added to reach a final SDS concentration of 1%; the samples were incubated at 50 ${}^{{}^{\circ}}$C for 15 min on a shaking thermal block. Next, lysis buffer containing 20% Triton X-100 was added to reach a final concentration of 2% for Triton X-100. The samples were then incubated on ice for 5 min; 10 µL of each of the samples was separated for analysis with western blot. The remaining portions of each sample were incubated overnight at 4 ${}^{{}^{\circ}}$C with 250 µg of Pierce streptavidin magnetic beads (S beads) that were preequilibrated with lysis buffer.

For experiment 2, the forebrains were also homogenized on ice using a glass/Teflon homogenizer with 3 mL of lysis buffer (50 mM Hepes, pH 7.5, 150 mM NaCl, 1 mM EDTA, 0.25 µL benzonase, EDTA-free cOmplete mini protease inhibitor mixture; 11836170001; Roche Diagnostics). The samples were then transferred to an Eppendorf tube, and lysis buffer with 10% SDS was added to reach a final SDS concentration of 1%; the samples were incubated at 50 ${}^{{}^{\circ}}$C for 15 min on a shaking thermal block. Triton X-100 was not added in this experiment. Ten microliters of each of the samples was separated for analysis with western blot. The remaining portions of each sample were incubated for 2 h at room temperature with 250 µg of Pierce S beads that were preequilibrated with the lysis buffer.

In both experiments, the S beads incubated with samples were then collected with a magnetic stand, and the supernatant was discarded. S beads were washed as follows: two times with 300 µL of wash buffer 1 (50 mM Hepes, pH 7.5, 150 mM NaCl, 1 mM EDTA, 2% SDS), two times with wash buffer 2 (50 mM Hepes, pH 7.5, 1% Triton X-100, 1% deoxycholate, 25 mM LiCl), two times with wash buffer 3 (50 mM Hepes, pH 7.5, 1 M NaCl), and five times with wash buffer 4 (50 mM ammonium bicarbonate). The bound proteins were then eluted by incubating the beads for 1.5 h at 60 ${}^{{}^{\circ}}$C with 100 µL of elution buffer (50 mM ammonium bicarbonate, 0.1% Rapigest SF surfactant, 2 mM biotin) followed by a second round or elusion with 100 µL of elution buffer for 1.5 h at 60 ${}^{{}^{\circ}}$C. The final protein concentration was measured by bicinchoninic acid (BCA) assay. Liquid chromatography and mass spectrometry was performed by the W. M. Keck Foundation Biotechnology Resource Laboratory, Yale School of Medicine, New Haven, CT.

### Statistical Analysis of the Identified Biotinylated Proteins.

To identify the proteins that were present at significantly different concentrations in the BioID2-synaptopodin samples relative to the BioID2-Shank3* samples, we performed statistical analysis. The material from each mouse brain was considered one biological replicate. The goal of this analysis was to correct for the variability between different samples in terms of the expression level of the BioD2 fusion protein and of the efficacy of the fusion protein in performing biotinylation. In experiment 1, we measured the enrichment of any given protein in each group as the ratio between the mean number of peptides in the BioID2-synaptopodin samples relative to BioID2-Shank3* samples. To determine the significance of enrichment, we performed a Student’s *t* test between the samples of the two groups and defined a *P* value < 0.1 as significant enrichment.

In experiment 2, we performed an additional normalization to correct for the variation in the yield of biotinylation between BioID2-synaptopodin and BioID2-Shank3* and among different animals. To this aim, we used the two major endogenously biotinylated proteins, propionyl-coenzyme A carboxylase alpha chain and pyruvate carboxylase, as an internal control. Higher numbers of reads for these endogenous proteins relative to proteins biotinylated by exogenous BioID2 meant lower biotinylation yield. Subsequently, we normalized the amount of the identified proteins for each sample to the sum of the number of reads for these two proteins, and the normalized values for synaptopodin and Shank3* were compared as described above for the first experiment.

For experiments 1 and 2, 64 and 67%, respectively, of the proteins identified as significantly enriched in the proximity of synaptopodin were also found in the proximity of synaptopodin in the other experiment. Such proteins are depicted in bold in *SI Appendix*, Fig. S2.

### Determining the Correlative Expression of Proteins Relative to Synaptopodin.

To find the genes with an expression pattern similar to that of synaptopodin, the number of reads per protein per mouse brain cell was obtained from Dropviz (42). An in-house R code was developed to calculate the Pearson correlation coefficient for individual genes with synaptopodin according to Eq. **1** (in the text). Due to the large sample size (*n* = 939, 489 cells) and according to Eq. **2**, we estimate that nonzero correlation coefficients can be considered any value above $10^{-3}$, as they will have a *t* of 0.99 in the Student’s *t* distribution:

[2] $$r=\frac{t}{\sqrt{n-2+t^{2}}},$$

where *n* is the number of samples, 2 is the degrees of freedom assuming a normal sample distribution, and *r* is the Pearson correlation coefficient.

### Primary Neuronal Culture and Transfection.

Hippocampuses of postnatal day 0-1 C57BL/6 mice (The Jackson Laboratory) were dissected on ice in Hibernate-A media (Thermo Fisher). Tissues were then washed in ice-cold dissociation media (5.8 mM MgCl_2_, 0.252 mM CaCl_2_, 10 mM Hepes, pH 7.4, 1 mM pyruvic acid, 81.7% Mg- and Ca-free Hank’s Balanced Salt Solution (HBSS) from Thermo Fisher in water) and immediately digested in cysteine-activated Papain solution (17 U/mL Papain from Worthington, 20 µg/mL DNase I from Sigma, 2 mg/mL of l-cysteine hydrochloride from Sigma in dissociation media) for 30 min at 37 ${}^{{}^{\circ}}$C. Papain was then inactivated with 10% fetal bovine serum (FBS) in dissociation media followed by washes in dissociation media and in Neurobasal-A (Thermo Fisher) supplemented with 2% B27 and 2 mM l-glutamax; 120,000 to 150,000 cells were plated on the glass bottom of Mattek plates in 150 µL of neuronal growth media (Neurobasal-A supplemented with 2% B27, 2 mM l-glutamax, 15% gilial enriched media, 10% cortical enriched media). Prior to plating the neurons, Mattek dishes were incubated 0.1 mg/mL poly-d-lysine in borate buffer for at least 4 h. Four to sixteen hours after plating, 2 mL of neuronal growth medium was added per plate; 0.5 mL of neuronal growth medium was added per dish every 3 to 4 d afterward. Hippocampal neurons were transfected on DIV11-13 using the CalPhos Mammalian Transfection kit (Takara) per the manufacturer’s instructions and fixed or imaged at DIV16-24.

### Nonneuronal Cell Cultures and Transfections.

COS-7 cells were obtained from ATCC. Cells were maintained at 37 ${}^{{}^{\circ}}$C in a humidified atmosphere at 5% CO_2_. COS-7 cells were grown in Dulbecco’s Modified Eagle Medium (DMEM) (Thermo Fisher Scientific) supplemented with 10% FBS, 100 U/mL penicillin, 100 mg/mL streptomycin, and 2 mM glutamax (Thermo Fisher Scientific). All cell lines were routinely tested and always free from mycoplasma contamination.

The cells were seeded on glass-bottom Mattek dishes at least 6 h before transfection. All transfections of plasmids used Lipofectamin 2000 (Thermo Fisher) to the manufacturer’s specifications for 16 to 24 h in complete media.

### Live Cell Imaging and Immunofluorescence.

Confocal imaging was performed using LSM880 or LSM800 (Carl Zeiss Microscopy) with a 63×/1.40-NA plan-apochromat differential interference contrast oil immersion objective and 32-channel gallium arsenide phosphide photomultiplier tube area detector; 405-, 488-, 561- and 633-nm laser lines were used in this study.

For live imaging, nonneuronal cells were imaged using live cell imaging buffer (Life Technologies), and cultured neurons were imaged in modified Tyrode buffer (119 mM NaCl, 5 mM KCl, 2 mM CaCl_2_, 2 mM MgCl_2_, 30 mM glucose, 10 mM Hepes, pH 7.35).

Halo ligand, JF646, was used at a final concentration of 200 nM. Cells were incubated with JF646 for 1 h, rinsed, and then, incubated for 30 min (all in culture medium) before imaging in live cell Imaging buffer.

For presentation purposes, brightness and contrasts of images were adjusted using the ImageJ software. Some of the high-magnification fields were enlarged using the Adjust Size function on ImageJ.

### Quantification of the Enrichment Factor for EGFP-Pdlim7 in Spines.

To quantify the enrichment of EGFP-Pdlim7 fluorescent signal in spines relative to dendritic shafts, images of six wild-type and six synaptopodin KO neurons expressing EGFP-Pdlim7 were thresholded, and a mask was generated of the brightest spots in dendritic spines of each neuron. The mean intensities of these detected spots were measured, and the average of the top 40 brightest spines per neuron, *i*, was calculated ($\mu_{\mathrm{spine},i}$). In addition, five regions of the dendritic shafts of each neuron were manually selected, and the mean intensity for those regions was also calculated ($\mu_{\mathrm{shaft},i}$). The enrichment factor for each neuron, *i*, was calculated using Eq. **3**:

[3] $$\mathrm{EnrichmentFacto}r_{i}=\frac{\mu_{\mathrm{spine},i}}{\mu_{\mathrm{shaft},i}}.$$

### Quantifying the Number of Spines with the ER and with the SA.

To determine the number of spines with the ER and with the SA, we used three different datasets of 3D volume EM images. The first, which had the highest resolution, was from the mouse cerebral cortex imaged by FIB-SEM, as reported in ref. 23. To minimize bias, PSDs were detected first, and the presence of the ER and SA was then determined in the 3D volume. Two datasets available on MICrONS Explorer, which covered a much larger area of the mouse brain and contained segmentation data for each neuron, were also analyzed. Five neurons were randomly selected in each of the two datasets, and for each neuron, the spines along a single dendrite were examined starting from the cell body and progressing toward the end of the dendrite. This minimized bias for selection of the spines examined.

### Antibodies and Reagents.

The list of antibodies, their working dilution, and the supplier for this study can be found in *SI Appendix*, Table S2. pAAV-HA-GFP-Pdlim7, pAAV-HA-Magi1, and pAAV-HA-Magi2 were cloned by Genscript. AAV2/9 packaging for pAAV-BioID2-synaptopodin was done by Janelia Virus Services, Howard Hughes Medical Institute, and packaging of pAAV-BioID2-Shank3* was done by Penn Vector Core, University of Pennsylvania. The following constructs were gifts: MCS-BioID2-HA from K. Roux, Sanford Research, Sioux Falls, SD (Addgene; plasmid 74224); pCDNA flag MAGI1c from W. Sellers, Harvard Medical School, Boston, MA (Addgene; plasmid 10714); Myc rat S-SCM from Y. Hata and Y. Takei (Addgene; plasmid 40213); EGFP-*α*-actinin-2 from J. Hell, University of California Davis, Davis, CA (Addgene; plasmid 52669); EGFP-MyosinIIA from M. Krummel, University of California San Francisco, San Francisco, CA (Addgene; plasmid 38297); F-Tractin-mCherry from T. Meyer, Weill Cornell, New York, NY (Addgene; 155218); pAcGFP-Sec61*β* from E. Schirmer, University of Edinburgh, Edinburgh, UK (Addgene; plasmid 62008); mRFP-synaptopodin from A. Triller Institut de Biologie de l’Ecole Normale Supérieure, Paris, France; and RFP-Shank3 from A. Koleske, Yale School of Medicine, New Haven, CT. pAAV-GFP-MCS and EGFP-VAPB were previously constructed in our laboratory.

Biotin was purchased from Sigma (B4501). Latrunculin A and Rock inhibitor Y-27632 were from EMD Millipore Corp.

### Generation of Plasmids.

Most constructs were generated with regular cloning protocols or through site-directed mutagenesis. Some constructs were ligated using Gibson assembly (NEB) or In-Fusion Cloning (Takara Bio). Details of primer sets, enzymes, techniques, and plasmids used for each construct can be found in *SI Appendix*, Table S3.

The desired open reading frame for Pdlim7 (GenBank accession no. AF345904.1) was amplified by PCR from Human Universal QUICK-Clone II (Takara; 637260) using the primers depicted in *SI Appendix*, Table S3 and inserted into the pEGFP-C1 plasmid through Gibson assembly.

All constructs were sequenced in their entirety before use in any experiment.

## Supplementary Material

Supplementary File

Supplementary File

Supplementary File

## Data Availability

All study data are included in the article and/or supporting information.
