# Supplementary material for: Proximity proteomics of synaptopodin provides insight into the molecular composition of the spine apparatus of dendritic spines
Source: Proc Natl Acad Sci U S A. 2022 Oct 10;119(42):e2203750119. doi: 10.1073/pnas.2203750119 (PMC9586327; doi:10.1073/pnas.2203750119)
Supplement: Supplementary File [file pnas.2203750119.sapp.pdf]

PNAS
www.pnas.org

Supplementary Information for

Proximity proteomics of synaptopodin provides insight into the molecular composition of the spine apparatus of dendritic spines

Hanieh Falahati, Yumei Wu, Vanessa Feuerer, Hans-Georg Simon and Pietro De Camilli

Pietro De Camilli.
E-mail: pietro.decamilli@yale.edu

This PDF file includes:

Figs. S1 to S4
Tables S1 to S3
Legend for Movie S1
Legend for Dataset S1

Other supplementary materials for this manuscript include the following:

Movie S1
Dataset S1

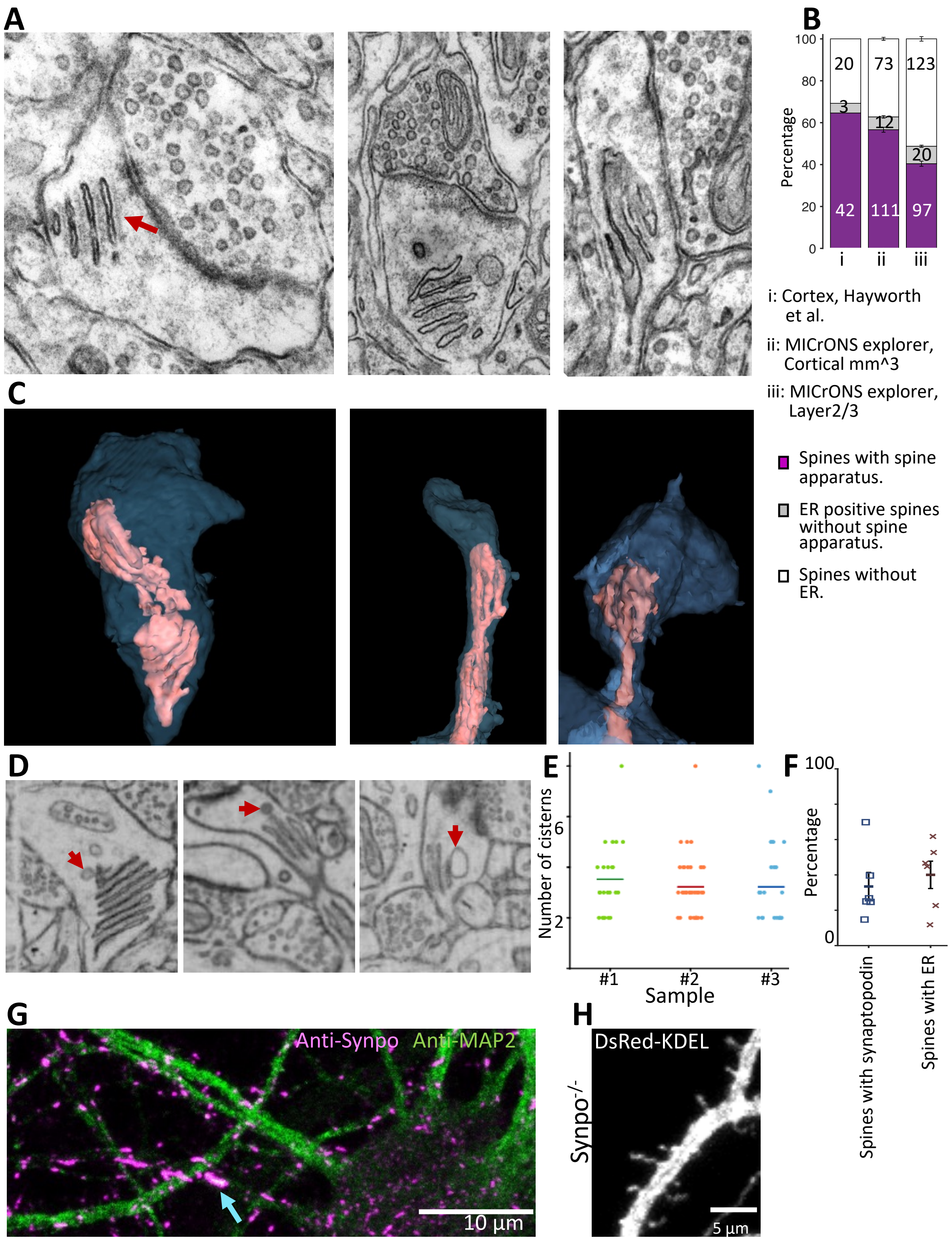

Fig. S1. Morphological features of the SA in neurons of the mouse cerebral cortex. **A.** Examples of SA as visualized by transmission EM. Red arrow shows the dense matrix at the free surface of an outer cistern. **B.** Quantification of the number of spines with spine apparatus (magenta), with ER but not spine apparatus (gray) and without ER (white). Samples analyzed are as follows: FIB-SEM volumes of cerebral cortex reported in Heyworth et al. (23) (i); 196 spines from five neurons in Cortex mm^3 (ii) and 240 spines from five neurons in layer2/3 (iii) datasets available on MICrONS explorer. Error bars in ii and iii show the standard deviation over mean for the five neurons in each of the two dataset. **C.** Examples of semiautomatically reconstructed dendritic spines and SAs from 3D volumes acquired by FIB-SEM. The ER is in red and the plasma membrane in blue. **D.** Examples of SAs in contact with tubulovescicular structures (red arrows). **E.** Number of cisterns per SA in three distinct samples of cerebral cortices (23). Each point represents a single SA, and the mean number of stacks for each sample is shown as a line. Data and images reported in **C-D** were extracted from FIB-SEM acquired 3D volumes published previously (23). **F.** Percentage of spines with synaptopodin, and spines with ER in cultured hippocampal neurons. Each point represent a single neuron. Means and he standard deviations are shown. **G.** In a subset of cultured hippocampal neurons, anti-synaptopodin staining shows the localization of this protein at axonal initial segment (blue arrow, note the lack of MAP2 staining). **H.** Presence of ER in dendritic spines of a cultured hippocampal neuron of a synaptopodin KO mouse expressing the ER marker DsRed-KDEL.

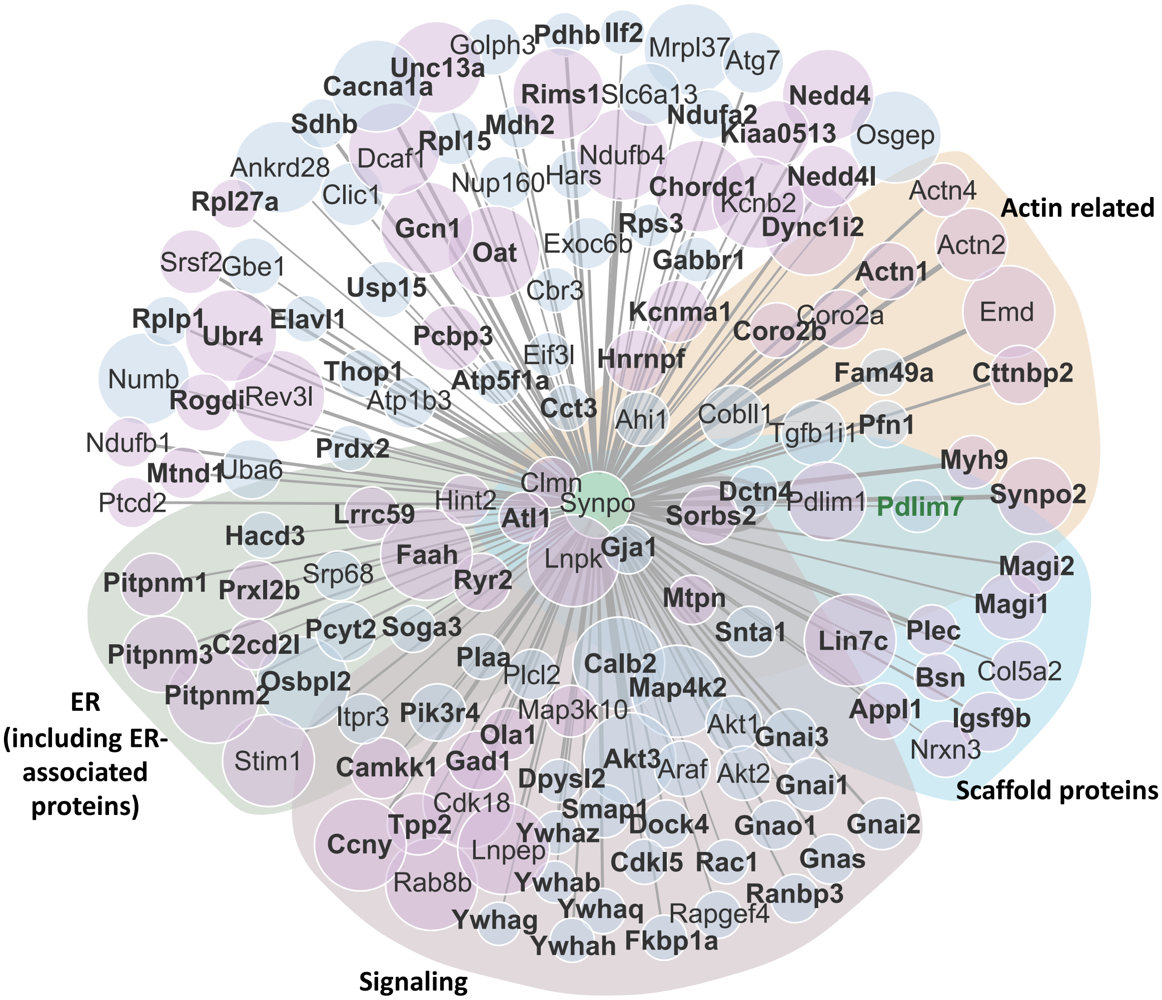

Fig. S2. Proteins identified as neighbors of synaptopodin by proximity labeling. Proteins identified in the first and second round of proximity biotinylation are shown in blue and purple circles, respectively. The size of the circles correlates with the enrichment in BioID2-Synpo samples relative to BioID2-Shank3* samples, and the size of the lines correlates with the significance of the enrichment $[-log(p_{value})]$. Proteins of different functional classes are grouped in different shades.

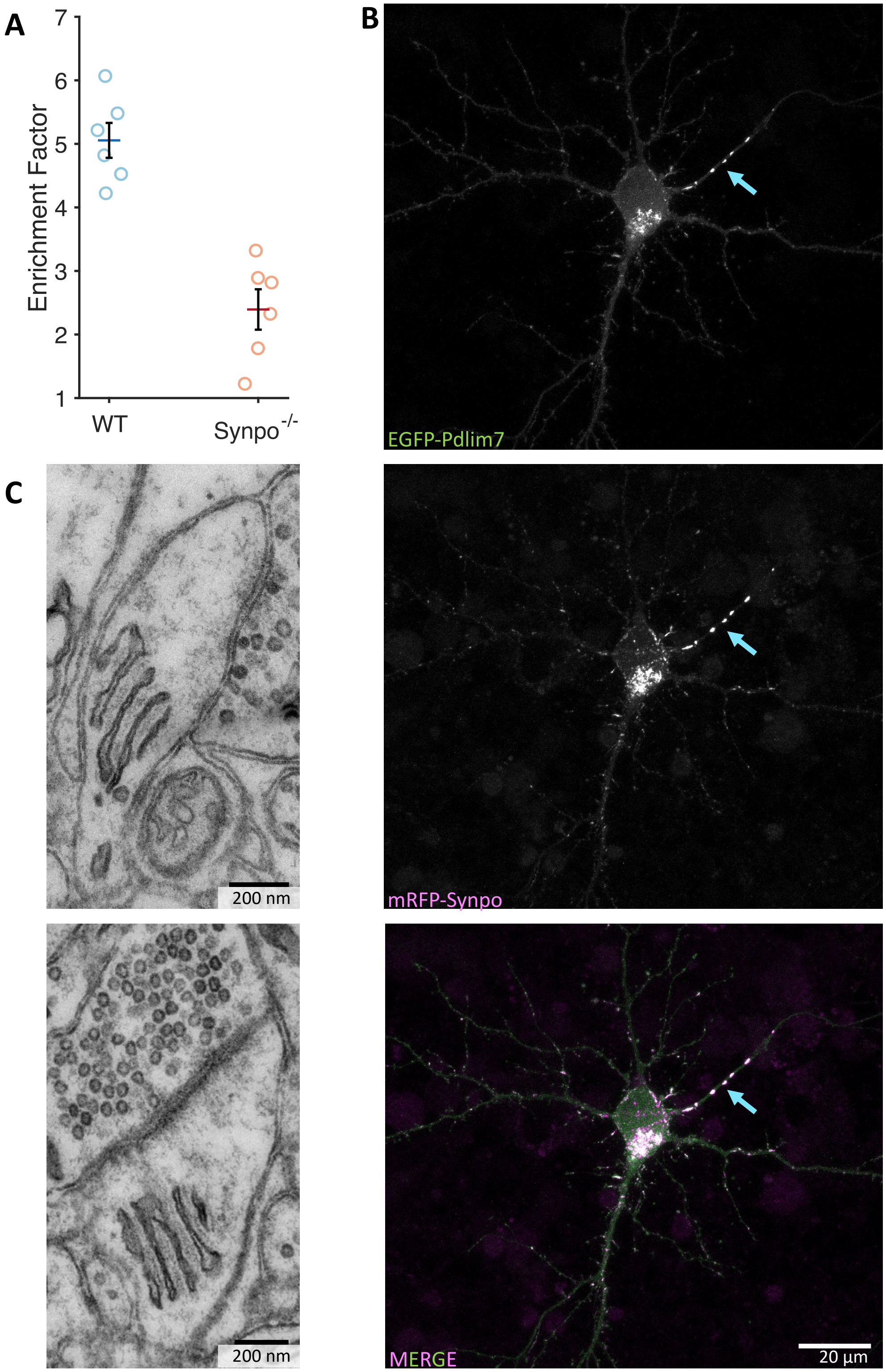

Fig. S3. Association between Pdlim7 and Synaptopodin in neurons. **A.** Enrichment factor for EGFP-Pdlim7 at the dendritic spines compared to dendritic shaft is calculated for wild-type (WT) and synaptopodin KO neurons. Each point represents a single neuron. **B.** Pdlim7 colocalizes with synaptopodin at axonal initial segment (blue arrow). **C.** Transmission EM of spine apparatus in Pdlim7 KO mice.

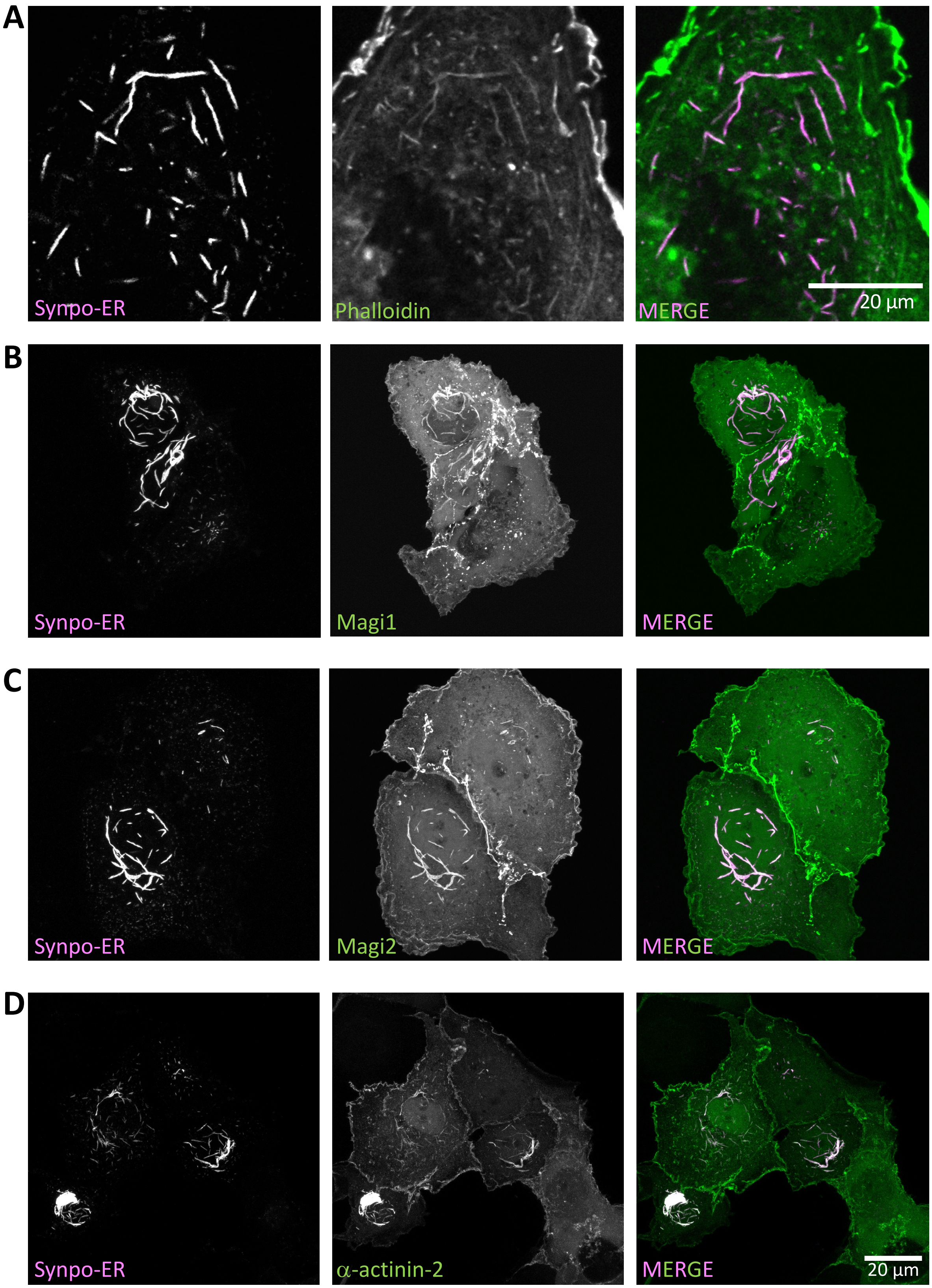

Fig. S4. Synaptopodin interacts with α-actinin-2, MAGI1 and MAGI2 in COS-7 cells. **A.** Expression of synaptopodin-ER in COS-7 cells results in the formation of large synaptopodin assemblies on the ER that contain filamentous actin as revealed by phalloidin. **B-D.** In these cells, coexpression of synaptopodin-ER (Synpo-mCherry-Sec61β) with EGFP- Magi1 (**C**), EGFP-Magi2 (**D**), or EGFP-α-actinin-2 (**B**) results in the recruitment of these proteins to assemblies of the ER- tethered synaptopodin.

Table S1. List of ER associated proteins examined for their interaction and colocalization with synaptopodin (Synpo).

Protein	Significantly enriched in the proximity of Synpo vs. Shank3	Found in the proximity of Synpo	Type of experiment	Localization	Reagent
Stim1	Yes	Yes	Coexpression in neurons with Synaptopodin, with or without Orai	Diffuse throughout the entire ER	Addgene plasmid #18857
LRRC59	Yes	Yes	Coexpression in COS7 cells and neurons with Synaptopodin	Clusters on ER in COS7 cells; not enriched in the spines	pEGFP-LRRC59 (this study), anti-LRRC59 (Sigma HPA030829)
Soga3	Yes	Yes	Coexpression in COS7 cells and neurons with Synaptopodin	Diffuse throughout the entire ER	pEGFP-Soga3 (this study)
Plekhf2	Yes	Yes	Coexpression in COS7 cells and neurons with Synaptopodin	Diffuse throughout the entire ER and on endosome-like structures in COS7 cells; In neurons enriched in the spines	Plekhf-EGFP (this study)
Calmin	Yes	Yes	Coexpression in COS7 cells with Synaptopodin	Diffuse throughout the entire ER	pEGFP-Calmin (this study)
Lnpk	Yes	Yes	Coexpression in COS7 cells and neurons with Synaptopodin	Diffuse throughout the ER in COS7 cells, Partial colocalization with synaptopodin in neurons	Addgene plasmid #86687
Faah	Yes	Yes	antibody staining	Everywhere (not sure of the antibodies specificity in neurons)	Abcam ab54615
Pitpnm1	Yes	Yes	Coexpression in HeLa cells and neurons with Synaptopodin	Cytosolic in HeLa, Partial overlap with Synpo in neurons	Construct from De Camilli lab
Pitpnm2	Yes	Yes	Coexpression in HeLa cells and neurons with Synaptopodin	Cytosolic in HeLa, diffuse throughout the entire neurons	Construct from De Camilli lab
Inf-2	No	Yes	Coexpression in COS7 cells with Synaptopodin	Diffuse throughout the entire ER.	GFP-Inf-2 with CAAX (gift from Henry Higgs lab)
Nesprin3	No	Yes	Coexpression in COS7 cells with Synaptopodin	No colocalization	Addgene plasmid #54203
VAPB	No	Yes	Coexpression in COS7 cells and neurons with Synaptopodin	Diffuse throughout the entire ER	Construct from De Camilli lab
MOSPD1	No	No	Coexpression in COS7 cells and neurons with Synaptopodin	Diffuse throughout the entire ER	Gift from Jacques Neefjes lab
MOSPD2	No	No	Coexpression in COS7 cells with Synaptopodin	Diffuse throughout the entire ER	Gift from Fabien Alpy lab
MOSPD3	No	No	Coexpression in COS7 cells with Synaptopodin	Diffuse throughout the entire ER	Gift from Jacques Neefjes lab
TMCC1	No	No	Coexpression in COS7 cells with Synaptopodin and Pdlim7, coexpression in neurons with Synpo	No colocalization with Synpo. Pdlim7 alone can be wrapped in TMCC1 assemblies, but this association is disrupted when Synpo is coexpressed.	Addgene plasmid #120931
TMCC2	No	No	Coexpression in COS7 cells with Synaptopodin	On ER, with some bright assemblies. No colocalization with Synpo	Addgene plasmid #121047
TMCC3	No	No	Coexpression in COS7 cells with Synaptopodin	On ER, with some bright assemblies. No colocalization with Synpo	Addgene plasmid #121048
CLIMP-63	No	No	Coexpression in COS7 cells and neurons with Synaptopodin	Clusters on ER, in neurons mostly in cell body. No colocalization with Synpo	Gift from Bewersdorf lab

Table S2. List of antibodies used in this study.

Protein	Company; Catalog number	Antibody species	Working dilution for immunocytochemistry	Working dilution for immunoblotting
Synaptopodin	Sigma S9442-200UL	Rabbit	1:1000	1:2500
BioID2	BioFront Tech BID2-CP-100	Chicken	1:1000	NA
Streptavidin-Alexa Fluor 647	Thermofisher S21374		1:1000	1:5000
HA	Sigma 12158167001	Rat	1:500	NA
Phalloidin-Alexa Fluor 488	Thermofisher A12379		1:300	NA

Table S3. List of constructs produced in this study.

Construct	Cloning method	Primers	Backbone	Template/ insert
CMV-BioID2-Synaptopodin	Digestion/ ligation with AgeI and BsrGI	Primer 1: AGCGCTACCGGTATGttcaagaacctgatctggctg<br>Primer 2: tccggacttgtacaagcttcttctcaggctgaactc	mRFP-Synaptopodin	MCS-BioID2-HA
pAAV-BioID2-Synaptopodin	Digestion/ ligation with NdeI and ClaI	Primer 1: GTGTATCATATGCCAAGTACG<br>Primer 2: agatctatcgatTAAGATACATTGATGAGTTTGGAC	pAAV-GFP-MCS	CMV-BioID2-Synaptopodin
pAAV-BioID2-Shank3	Digestion/ ligation with BglII and EcoRI	Not applicable.	pAAV-BioID2-Synaptopodin	mRFP-Shank3
pAAV-BioID2-Shank3* (1055-1806)	Digestion/ ligation with HindIII	Not applicable.	pAAV-BioID2-Shank3	
pEGFP-Pdlim7	Gibson Assembly	Primer 1: ggactcagatctcgagctcaggattccttcaaggtagtgctgg<br>Primer 2: ccgtcgactgcagaattcgatcacacatgagagaaggcatggc	pEGFP-C1	Gene bank ID: AF345904.1
pEGFP-Pdlim7-PDZ domain	PCR, Digestion / ligation	Primer 1: GAGCTCAAGCTTTGGATTCCTTCAAGGTAGTGCTGG<br>Primer 2: TCCGGTGGATCCTTACTGGGCCCTGCTGAGGCC	Not applicable.	pEGFP-Pdlim7
pEGFP-Pdlim7-Linker only	PCR, Digestion / ligation	Primer 1: gcccgccgatcgtaaGTGAtcgaattctgcagtcgacgg<br>Primer 2: gcccgccgatcgGGGAGTCTTGCCGTTGTTGC	Not applicable.	pEGFP-Pdlim7
pEGFP-Pdlim7-3 LIM domains	PCR, Digestion / ligation	Primer 1: gcccgccgatcgGTGTGTCACCAGTGCCACAAGGTC<br>Primer 2: gcccgccgatcgagctcgagatctgagtccgga	Not applicable.	pEGFP-Pdlim7
pEGFP-Pdlim7 ΔPDZ domain	PCR, Digestion / ligation	Primer 1: agctcgagatctCCGGTTCAGAGCAAACCGC<br>Primer 2: agctcgagatctgagtccgga	Not applicable.	pEGFP-Pdlim7
pEGFP-Pdlim7 ΔLinker region	PCR, Digestion / ligation	Primer 1: gcccgccgatcgGTGTGTCACCAGTGCCACAAGGTC<br>Primer 2: gcccgccgatcgCTGGGCCCTGCTGAGGCC	Not applicable.	pEGFP-Pdlim7
pEGFP-Pdlim7 Δ3LIM domains	PCR, Digestion / ligation	Primer 1: gcccgccgatcgtaaGTGAtcgaattctgcagtcgacgg<br>Primer 2: gcccgccgatcgGGGAGTCTTGCCGTTGTTGC	Not applicable.	pEGFP-Pdlim7
pEGFP-Magi1	PCR, Digestion / ligation	Primer 1: GAGCTCAAGCTTattcgaaagtgatccagaagaagaac<br>Primer 2: GCCCGCGGTACCtcagatgctgaggtcggtg	pEGFP-C1	pCDNA flag MAGI1c
pEGFP-Magi2	PCR, Digestion / ligation	Primer 1: GCTCGAGGAATTCaACTACCatgtccaaaagcttg<br>Primer 2: GCCCGCGGTACCctacttccggcaggcctg	pEGFP-C1	Myc rat S-SCAM
pAAV-BioID2-Pdlim7	PCR, Digestion / ligation	Primer 1: ccgtcgactgcagaattcgatcacacatgagagaaggcatggc<br>Primer 2: gagaagaagcttggattccttcaaggtagtgctgg	pAAV-BioID2-Shank3*	pEGFP-Pdlim7
Synpo-ER (Synpo-GFP-Sec61β)	InFusion	Primer 1: cgctagcgctaccggatggaggggtactcagaggaagc<br>Primer 2: catggtggcgaccggtacctgatcccccactaccgccagacctccctt gaagcagaaggagggcttcc	GFP-Sec61β	mRFP-Synpo
Synpo-ER (Synpo-mCherry-Sec61β)	Digestion-Ligation with BsrG1 and AgeI	Not applicable.	Synpo-GFP-Sec61β	pmCherry-C1

Movie S1. Part of a dendrite reconstructed using an in house semiautomatic algorithm from FIB-SEM images of mouse cortical neurons reported previously (?). Plasma membrane is shown in blue, post-synaptic density in yellow, and ER in red.

SI Dataset S1 (dataset_one.txt)

Relative abundance of proteins (abundance in BioID2-synaptopodin/ abundance in BioID2-Shank3*) significantly enriched in the proximity proteome of either synaptopodin or Shank3*, and p-Value of the enrichment assessed by Students t-test.
